# Precise delivery and controlled release: strategies and advances in TLR7/8 agonist prodrugs for cancer immunotherapy

**DOI:** 10.3389/fimmu.2026.1791263

**Published:** 2026-02-26

**Authors:** Yutao Zou, Ting Li, Huoying Pan, Jiangle Liu, Jingxuan He, Haoyu Ju, Weiqi Wang, Xiaohua Zheng

**Affiliations:** 1 The People’s Hospital of Danyang, Affiliated Danyang Hospital of Nantong University, Danyang, China; 2 School of Pharmacy, Nantong University, Nantong, Jiangsu, China

**Keywords:** immune adjuvant, immunotherapy, prodrug strategy, resiquimod, TLR7/8 agonists

## Abstract

Toll-like receptor 7/8 (TLR7/8) agonists (such as resiquimod-R848) are potent immune adjuvants. However, their clinical use is limited by severe systemic toxicity. To address this challenge, prodrug strategies have emerged as a key solution. In recent years, researchers have developed various prodrugs through chemical modifications. These prodrugs can be selectively activated within the tumor microenvironment in response to specific triggers, such as hypoxia, ultrasound, radiotherapy, or overexpressed enzymes. This approach enables spatiotemporally controlled release of the active drug and significantly reduces systemic inflammatory responses. To further enhance therapeutic efficacy and targeting precision, advanced delivery systems (protein nanoparticles, polymeric nanogels, liposomes, and nanoparticle suspensions) have been employed to carry these prodrugs. Such systems not only provide sustained release but also allow co-delivery of antigens, siRNA, or chemotherapeutic agents. This facilitates synergistic modulation of the tumor immune microenvironment. When combined with immune checkpoint inhibitors (ICIs) or chemotherapy, they exhibit strong synergistic antitumor effects and induce durable immune memory. Notably, several of these approaches have already entered clinical evaluation. By summarizing recent advances in both prodrug chemistry and sophisticated delivery platforms, this review highlights a promising path toward precise and controllable delivery of TLR7/8 agonists. We hope this integrated strategy will pave the way for safer and more effective cancer immunotherapies.

## 1 Introduction

Malignant tumors have long posed a serious threat to human health, prompting extensive research into various therapeutic strategies, including chemotherapy (1–5), radiotherapy (6–9), phototherapy (10–15), and immunotherapy (16–19). Among these treatment modalities, the rise of cancer immunotherapy has brought a revolutionary shift in cancer treatment (20, 21). A key strategy in this field is to activate the innate immune system to kick-start and boost adaptive antitumor immune responses (22–30). Among the molecules employed for this purpose, imidazoquinoline (IMDQ) derivatives, particularly resiquimod (R848), a potent Toll-like receptor 7/8 (TLR7/8) agonist, have emerged as central players (31–37). R848 strongly binds to TLR7/8 receptors inside endosomes of antigen-presenting cells (APCs), such as dendritic cells (38–41). It then triggers the MyD88 signaling pathway, leading to robust production of type I interferons, tumor necrosis factor-α (TNF-α), and other pro-inflammatory cytokines (42–46). This drives APC maturation and increases expression of co-stimulatory molecules (47–53). In recent years, R848-based nanotherapeutic systems have been extensively investigated (54–59). More importantly, R848 can reshape the immunosuppressive tumor microenvironment (TIME) (60). For instance, it reprograms pro-tumor M2-like tumor-associated macrophages (TAMs) into anti-tumor M1 types and converts myeloid-derived suppressor cells (MDSCs) into cells that can present antigens (61, 62). These changes create a favorable setting for cytotoxic T cells to become activated and infiltrate tumors. Because of these effects, R848 is an excellent adjuvant for therapeutic cancer vaccines and for reversing immune suppression.

However, R848’s potent immune-stimulating activity is hampered by its poor pharmacokinetic properties, a major clinical challenge (63–65). With a small molecular weight of just 314 Da, R848 quickly spreads from the injection site into the bloodstream after local administration (66–69). This uncontrolled systemic distribution causes off-target activation of immune cells throughout the body, leading to serious dose-limiting toxicities such as high fever, chills, and low lymphocyte counts (70–77). Repeated dosing may even cause immune tolerance, reducing long-term effectiveness (78–83). The clinical application of R848 is currently limited primarily to topical gels for the treatment of cutaneous lesions, and its significant barriers to systemic administration severely restrict its use in treating deep-seated or metastatic solid tumors (84, 85). This limitation has led researchers to explore prodrug strategies. The idea is simple: reversibly mask R848’s essential active group, mainly its aromatic amine, so the molecule stays inactive (“silent”) during delivery (84, 86). Only when it reaches the tumor and encounters specific disease-related signals or external triggers does it become active again. The goal is precise control in both time and space: confining strong immune stimulation strictly to the tumor site. This maximizes therapeutic benefit while minimizing or eliminating systemic side effects (61).

Significant progress has been made in designing “smart” prodrugs of R848 and its analogs, such as IMDQ. These designs add a “chemical switch” that can be cleaved only under tumor-specific conditions. Two main approaches have emerged. First are prodrugs activated by internal tumor signals. For example, many solid tumors are hypoxic. Scientists have masked R848’s amine as an azide group (R848-N_3_), which is selectively reduced back to the active form by upregulated cytochrome P450 enzymes in low-oxygen zones (84). Another design links IMDQ to a platinum(IV) prodrug via a γ-glutamyl linker. Inside tumor cells, glutathione first reduces the platinum part, and then membrane-bound γ-glutamyl transpeptidase cuts the linker to release active IMDQ, combining chemotherapy and immune activation in one step (62). Other strategies use the acidic pH of endosomes (via hydrazone bonds) or the highly reducing environment inside tumor cells (via disulfide bonds) to trigger drug release precisely where needed (87, 88). Second are prodrugs activated by external physical stimuli, offering unmatched spatiotemporal control. For instance, IMDQ-N_3_ can be injected systemically and then activated only in the tumor area using ultrasound (89). The free radicals generated by ultrasound cavitation efficiently reduce the azide to the active drug, but only within the treated region. Even more innovative is a radiation-activated approach: adding a single oxygen atom to R848 reduces its activity by thousands of times (86). When radiotherapy is applied to the tumor, it removes this “protective group,” restoring R848’s immune-stimulating power exactly where it’s needed, while the rest of the body remains unaffected (86). These clever chemical designs turn R848 from an uncontrollable state into a precision weapon unlocked only by the right key.

Yet even the best molecular design needs advanced delivery systems to reach its full potential. These systems improve pharmacokinetics, enhance tumor targeting, and add new therapeutic functions. Nanocarriers, such as liposomes and polymeric nanoparticles, take advantage of the enhanced permeability and retention (EPR) effect to passively accumulate in tumors. This boosts local drug concentration while reducing systemic exposure (89). For example, coupling R848 to α-tocopherol creates a lipid-friendly prodrug. When formulated with hyaluronic acid into a nanosuspension, it forms a long-lasting depot at the injection site after subcutaneous delivery, providing steady immune stimulation without leaking into circulation (71). For intravenous use, surface engineering enables active targeting. Mannose-coated nanoparticles, for instance, “hitch a ride” on albumin to specifically collect in tumor-draining lymph nodes (TDLNs), the command centers of immune response (90). In intratumoral delivery, ultra-long-acting release is critical. Using TransCon technology, R848 is linked via a hydrolyzable bond to hydrogel microparticles (91). A single injection can sustain effective drug levels in the tumor for weeks, greatly improving safety and convenience. Beyond delivery, these systems serve as multifunctional platforms. They can carry multiple immune-modulating agents at once to create synergy. A classic example is co-delivering antigen and R848 prodrug in the same particle, such as in computationally designed protein nanocages or cationic liposomes (92). This ensures both are taken up by the same APC, powerfully driving antigen-specific T-cell responses, which is essential for effective cancer vaccines. Even more advanced systems integrate several immune instructions. One “DC nanoregulator” combines IMDQ prodrug, siRNA targeting PD-L1 and mannan (a TLR4 agonist) (90). This simultaneously activates dendritic cells and breaks immune tolerance. In another approach, a covalent organic framework (COF) loaded with Fe(II) catalyst accumulates in tumors and uses bioorthogonal chemistry to activate both a doxorubicin prodrug and IMDQ at the same time, triggering immunogenic cell death and adjuvant effects together (93). Such engineering transforms isolated prodrug molecules into integrated, multifunctional therapeutic systems.

The true power of these prodrug-based strategies shines when combined with other therapies, and they are already moving toward the clinic. When paired with immune checkpoint inhibitors, they show strong synergy. Local immune activation and T-cell influx “heat up” cold tumors, creating the ideal environment for ICIs to work. In animal studies, R848-N_3_ plus anti-PD-1 antibody led to remarkable tumor control and even cures (84). Similarly, nanoparticle-loaded prodrugs combined with anti-CTLA-4 or anti-PD-L1 antibodies also showed enhanced effects (61). Notably, a TransCon-based TLR7/8 agonist is now in clinical trials alongside pembrolizumab (anti-PD-1), a major step toward real-world use (91). Combining with radiotherapy or chemotherapy reveals even deeper integration. Radiation-activated prodrugs rely on radiotherapy itself: as radiation kills tumor cells and releases antigens, it also switches on the immune adjuvant right there (86). This triggers antitumor immunity not only at the primary site but also at distant metastases, the so-called abscopal effect (86). Meanwhile, prodrugs like Pt-Glu-IMDQ release both cisplatin (a chemo drug) and IMDQ from a single molecule, delivering direct tumor killing and immune activation together, and establishing long-lasting immune memory (62). These combination strategies have shown outstanding results in preclinical models. They suggest that R848-based prodrug delivery could finally overcome toxicity barriers and become a new generation of safe, effective cancer immunotherapy.

**Figure 1:**
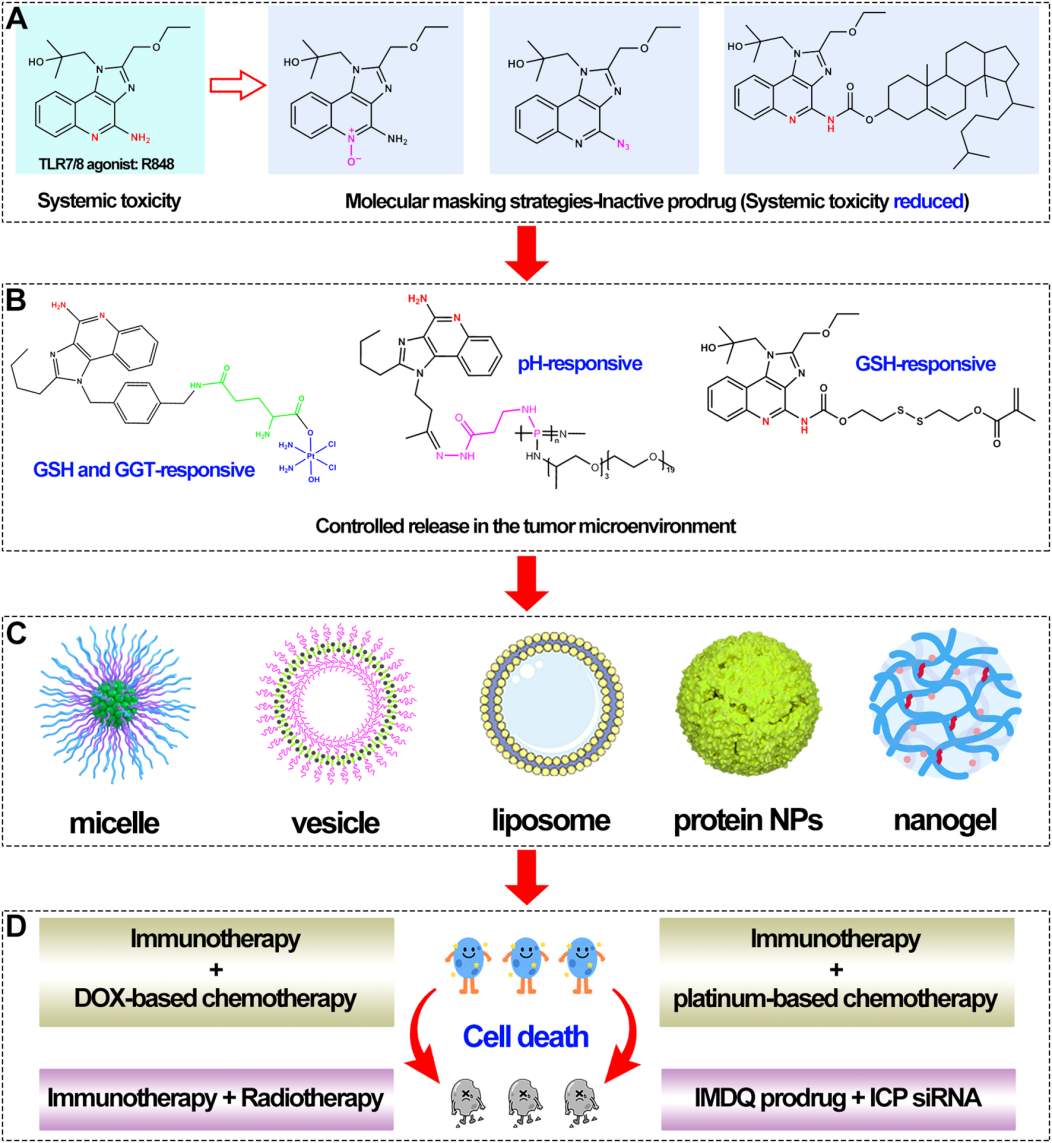
**(A)** Molecular marking strategies for toll-like receptor 7/8 agonists. **(B)** TME-responsive mechanism of various TLR7/8 agonists. **(C)** Various carrier nanoplatforms. **(D)** Combination therapy modalities.

In summary, this review covers recent advances in three interconnected areas: prodrug engineering of R848, intelligent delivery systems, and combination therapies. The field has evolved from simple molecular masking to avoid toxicity (**Figure 1A**), to smart prodrugs with active targeting and controlled release (**Figure 1B**), to multifunctional nanoplatforms that integrate multiple components (**Figure 1C**), and finally to systemic immune reprogramming through coordinated therapeutic programs (**Figure 1D**). The goal of this review is clear: First, to explain the chemical logic and activation mechanisms behind R848 prodrugs. Second, to review how different delivery systems improve targeting, duration, and functionality. Third, to highlight the synergistic potential, and underlying mechanisms, when these strategies are combined with radiotherapy, chemotherapy, or checkpoint inhibitors. Finally, to discuss current challenges, such as tumor heterogeneity affecting activation efficiency and long-term safety of carriers, and to outline future directions. We hope this review provides a clear, comprehensive picture of R848-based prodrug immunotherapy, and serves as a useful guide for future research and development.

## 2 Evolution and synergistic framework of R848 prodrug delivery

The clinical translation of resiquimod (R848) has been stalled by a fundamental conflict: its powerful but indiscriminate immune activation clashes with the goal of precise, tumor-targeted immunotherapy (61). Past approaches mostly relied on physical encapsulation or simple slow-release methods to merely “delay” systemic spread, a passive and limited tactic. In contrast, the advances highlighted in this review mark a true paradigm shift: from passive release to active programming. At its core, this new approach no longer treats R848 as a constantly “on” molecule that must be restrained. Instead, it re-engineers R848 into an intelligent therapeutic module that can be “awakened” only by specific instructions. This transformation is achieved through a three-tiered cascade of precision control.

**Figure 2:**
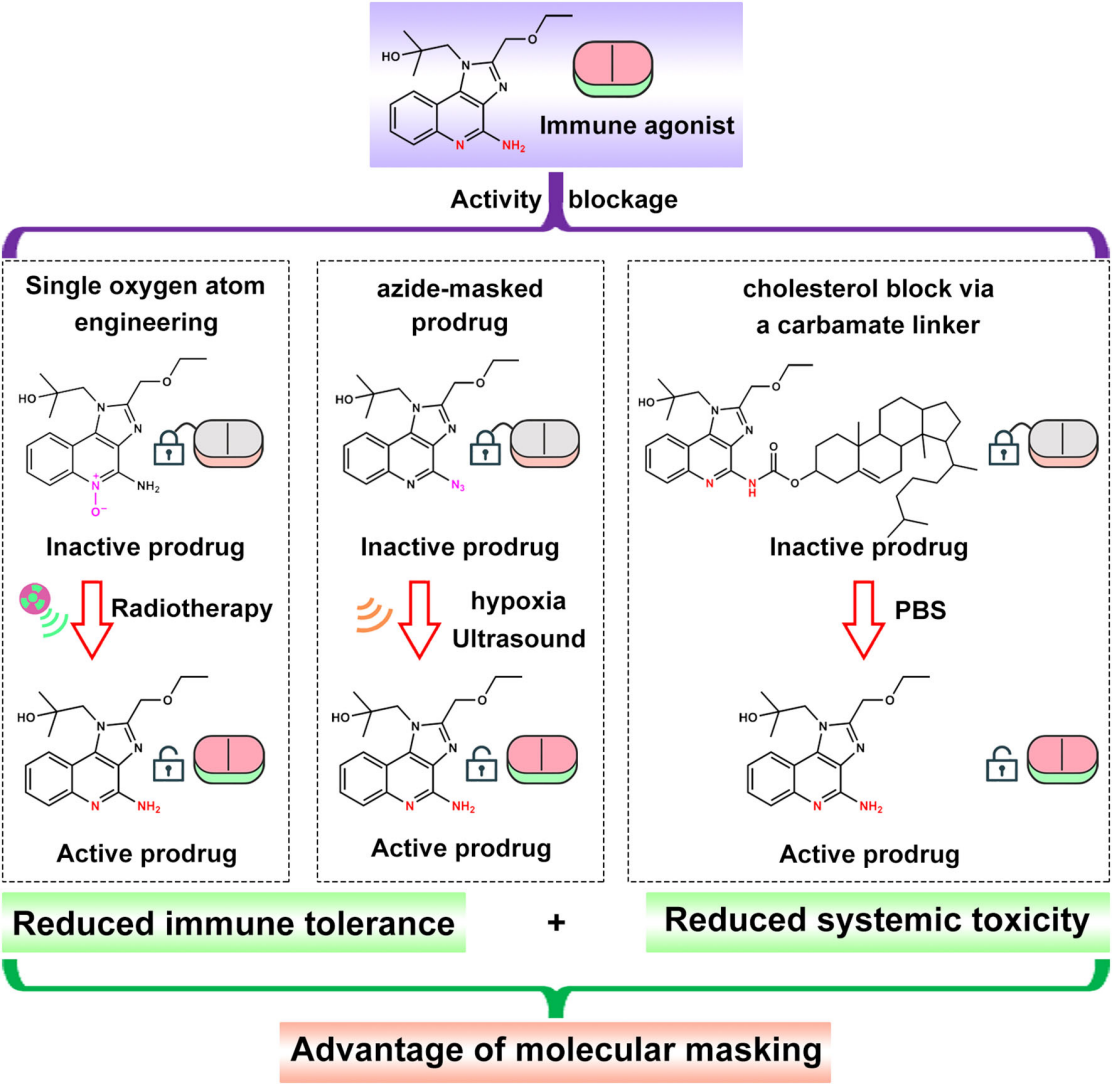
Activity blockage strategies of R848 molecular and the advantage of molecular masking.

Tier 1 is encoding molecular instructions. This is the chemical foundation of active control. Researchers chemically mask R848’s key active site, the aromatic amine, with cleavable “protecting-linker” groups (**Figure 2**). These groups are designed to respond only to specific cues found in tumors, such as hypoxia (84), overexpressed enzymes (62), or acidic pH (87), or to external triggers like ultrasound or radiotherapy. In effect, the drug is fitted with a smart lock that opens only when the right “key” is present. Only then is active R848 released. This directly links drug activity to spatial and temporal signals, enabling an on/off switch at the molecular level, far beyond what traditional carriers can offer.

Tier 2 is optimizing delivery logistics and integrating functions. Even the smartest molecular instruction needs an efficient delivery system to carry it out. Here, nanocarriers evolve from simple “delivery trucks” into multifunctional “mobile command centers.” Their job is twofold. First, they ensure the prodrug reaches its destination, whether the tumor or immune hubs like lymph nodes, with high efficiency and selectivity. Second, and more importantly, they serve as platforms to co-deliver multiple therapeutic “instructions.” For example, a single nanoparticle might carry both an R848 prodrug (to “activate dendritic cells”) and siRNA (to “lift immune suppression”) (61, 92). Because both agents enter the same antigen-presenting cell together, their effects are synchronized, creating synergy greater than the sum of its parts. This turns isolated immune stimulation into coordinated immune reprogramming.

**Figure 3:**
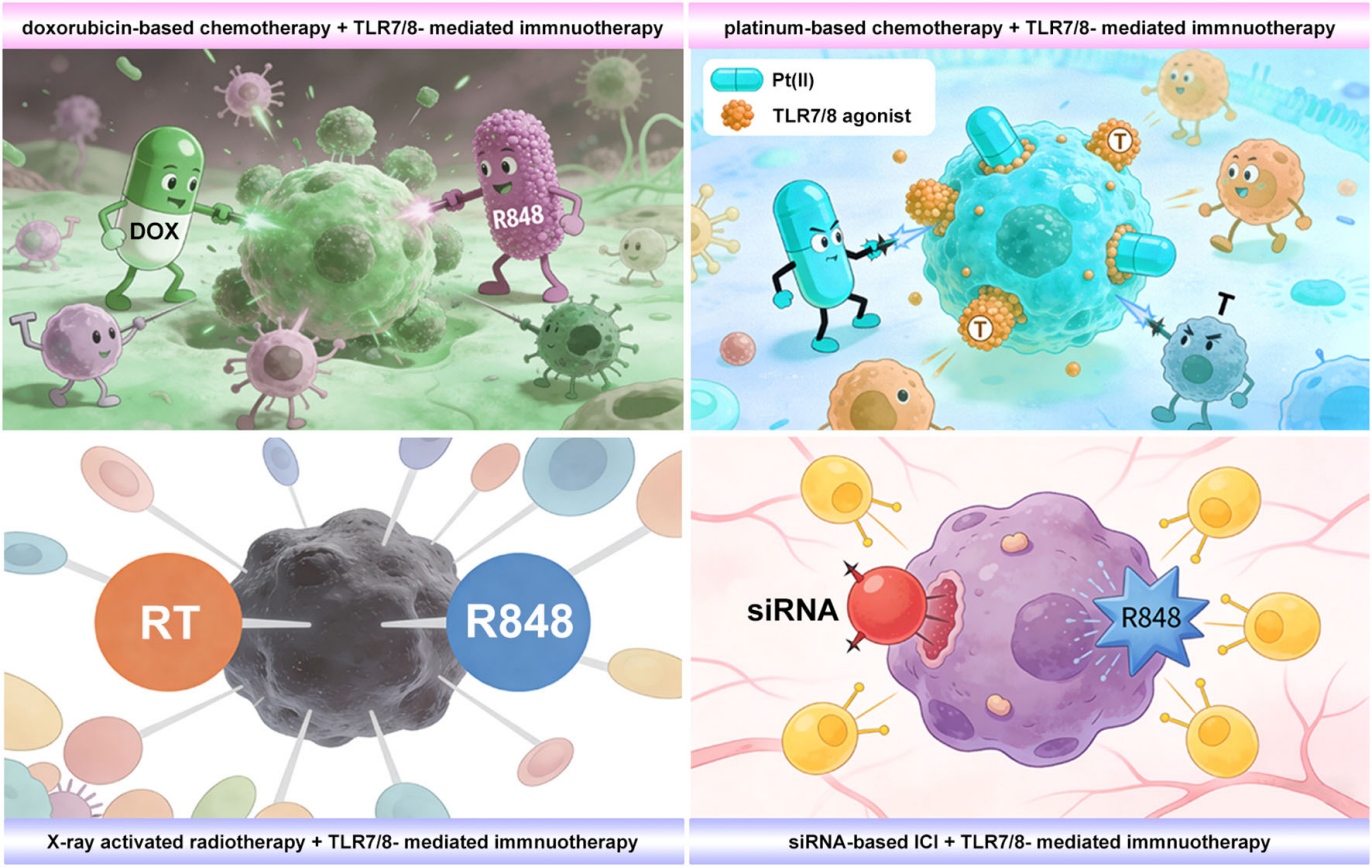
Schematic illustration of TLR7/8 agonists in combination with various therapeutic modalities for antitumor treatment.

Tier 3 is systematic deployment and therapeutic synergy. This top tier operates on two axes: vertical integration and horizontal coordination (**Figure 3**). On one hand, treatment mechanisms are linked in a programmed sequence. For instance, R848 prodrugs are combined with chemotherapeutics like cisplatin or doxorubicin, either at the molecular level or within a single nanoparticle. This creates a precise chain of events: chemotherapy kills tumor cells and releases antigens, while the simultaneously activated R848 boosts antigen presentation and adjuvant signaling. By aligning these steps in time and space, the strategy transforms what is normally an immunosuppressive chemo process into an effective *in situ* cancer vaccine (62, 93). On the other hand, distinct immune pathways are activated in parallel. The most successful example pairs R848-based innate immune activation with checkpoint blockade (61, 84). The R848 prodrug turns “cold” tumors “hot” by recruiting and priming T cells. Meanwhile, the checkpoint inhibitor removes the “brakes,” allowing those T cells to attack tumors effectively and form long-lasting memory. This dual-pronged approach overcomes resistance seen with single therapies and has produced strong abscopal effects and durable protection in multiple preclinical models (84, 91).

To better illustrate the core conceptual framework of this review and highlight the key advances in the cited studies, we have compiled a summary in **Table 1**. This table outlines the material design, performance characteristics, and major advantages of selected representative works. By organizing these details side by side, we aim to show not only how each system was engineered, but also why it stands out, whether through smarter activation, improved targeting, or stronger antitumor immunity.

**Table 1:** Nanoplatforms based on TLR7/8 agonist prodrugs for immunotherapy-integrated multimodal cancer treatment.

Material	Treatment/prodrug strategy		Property and therapeutic advantages	Ref
	Treatment	Prodrug strategy		
NL(pro-TLR7/8), resiquimod, doxorubicin	TLR7/8 agonist, ICIs, chemotherapy	cholesterol-conjugated	size≈123.38 ± 5.68 nm, 23 ± 2.5 mV, slow release in PBS, B16F10-OVA melanoma model	(61)
R848-N_3_, resiquimod	immunotherapy	azide-masking	hypoxic condition, CYP450 enzyme and NADPH, 4T1 breast carcinoma model	(84)
imidazoquinoline-N_3_	immunotherapy	azide-masking	size≈81.5 nm, zeta= −3.3 ± 0.8 mV, ultrasound-responsive, CT26	(89)
O-R848, resiquimod	RT, immunotherapy	Single atom engineering	radiotherapy-activated prodrug, MC38 bearing mice, 6 Gy, abscopal effect	(86)
Pt-Glu- imidazoquinoline	immunotherapy, chemotherapy, ferroptosis	small-molecule conjugation	GSH-responsive, GGT-responsive, 4T1, MDA-MB-231	(62)
ImQ-polymer, imidazoquinoline	immunotherapy	polymer conjugation	pH-responsive, endosomal prodrug degradability,	(87)
pResi, resiquimod	immunotherapy	small-molecule conjugation	protein NPs as carrier, physiological release	(92)
PEG-GLn- imidazoquinoline	immunotherapy	polymer conjugation	vesicles, Size≈195 nm, endosomal enzyme-responsive,	(94)
Resiquimod -Toco/HA-Toco	immunotherapy	small-molecule conjugation	nanosuspension, Size≈525 ± 8 nm, hydrolysis release of ester bond	(71)
DNR, imidazoquinoline	TLR4 agonist, TLR7/8 agonist, ICP siRNA	polymer conjugation	CatB cleavable linker, TDLNs modulation, B16F10	(90)
R848-Gel, resiquimod	immunotherapy	small-molecule conjugation	nanogel, GSH-responsive, diameter≈100 nm, zeta = −22.23 ± 2.74 mV, 4T1	(88)
TransCon TLR7/8 agonist, resiquimod	TLR7/8 agonist, anti-PD-1	polymer conjugation	Intratumoral delivery, polymeric hydrogel microspheres, CT26 tumor	(91)
CFe-FA, doxorubicin, imidazoquinoline	chemotherapy, immunotherapy	aryl azide carbonate unit-masking	Fe(II) catalysts-activated pro-DOX, bioorthogonal-activated pro-IMQ, 4T1	(93)

ICIs, immune checkpoint inhibitors; RT, radiotherapy; TDLNs, tumor-draining lymph nodes; pro-IMQ, a TLR7/8 agonist prodrug; ICP, inhibitory immune checkpoint; GGT, γ-glutamyltranspeptidase; DNR, DC-mediated nano-regulator.

## 3 Overcoming TLR7/8 agonist challenges with prodrugs

**Figure 4:**
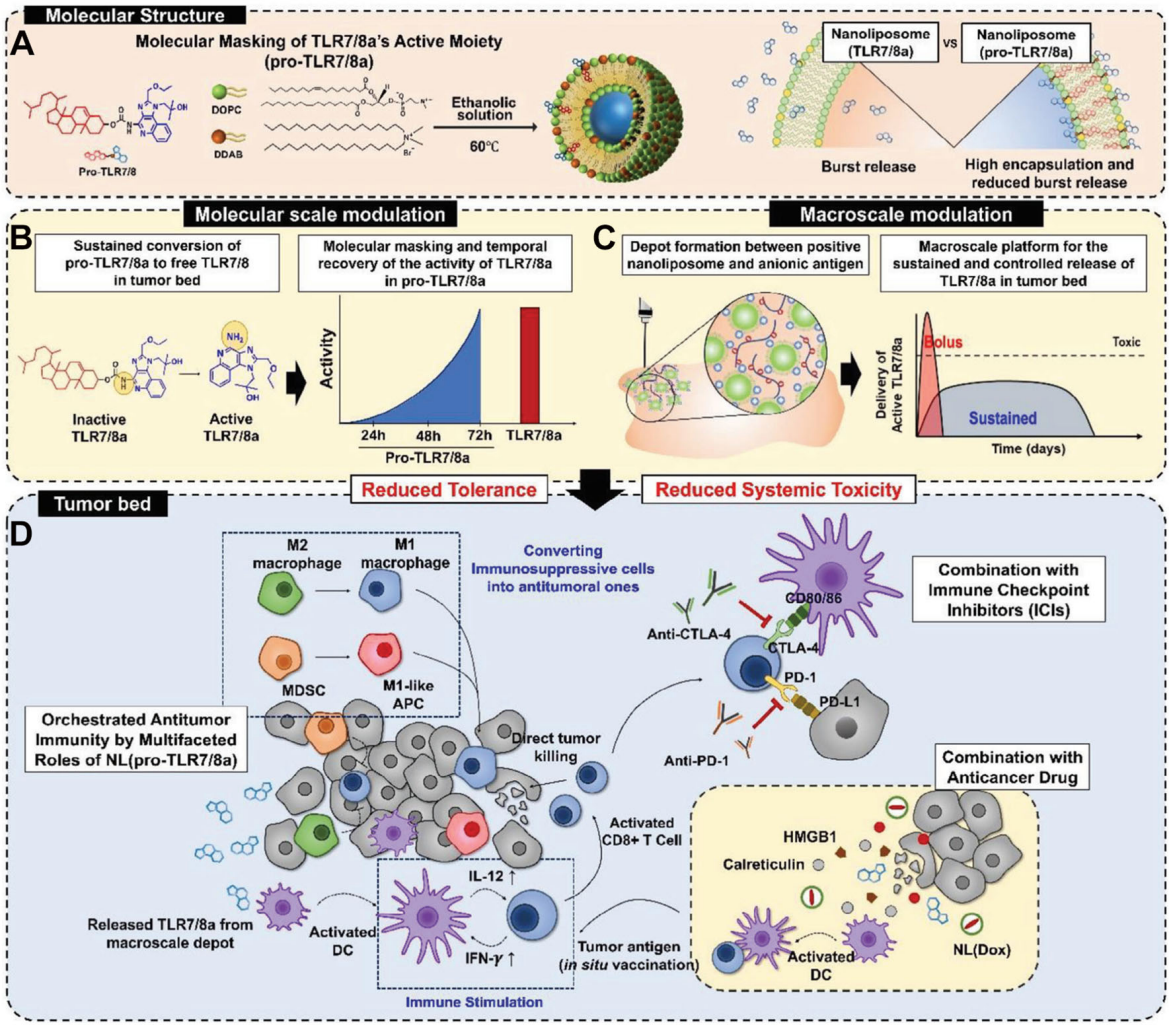
**(A)** Molecular structure of pro-TLR7/8a. **(B)** Activation strategy for pro-TLR7/8a. **(C)** Sustained and controlled release of pro-TLR7/8a at the tumor site. **(D)** Schematic illustration of pro-TLR7/8a-mediated antitumor immune responses. Reproduced with permission from (61). Copyright (2024), Wiley-VCH GmbH.

Toll-like receptor 7/8 agonists (TLR7/8a) hold great promise in cancer immunotherapy. However, their uncontrolled immune stimulation often leads to severe systemic toxicity. This side effect can completely offset any therapeutic benefit from direct administration. To address this issue, rational structural modification, specifically, the development of prodrug forms, has become a key strategy to enable clinical use. A growing body of research shows that the biological activity of R848 and similar compounds mainly depends on their aromatic amine group. Building on this insight, Lim and colleagues designed a prodrug by linking R848 to cholesterol via a carbamate bond, creating a TLR7/8a prodrug (pro-TLR7/8) (**Figure 4A**) (61). This linker can be selectively cleaved inside cancer cells, releasing active R848 precisely where it is needed. Thanks to the lipid-loving nature of cholesterol, this prodrug offers two major advantages. First, it significantly reduces the systemic toxicity associated with free R848. Second, it can be efficiently encapsulated into liposomes, enabling stable delivery and controlled release specifically in the tumor microenvironment (**Figures 4B, C**). When combined with doxorubicin-containing formulations, this system produces strong synergy between chemotherapy and immunotherapy (**Figure 4D**). It directly tackles the main bottleneck in translating potent TLR7/8 agonists like R848 into the clinic: severe off-target immune activation and systemic toxicity. Overall, this work demonstrates that molecularly engineered prodrugs are not just an improvement, they are a critical step toward safe and effective therapeutic use of TLR7/8 agonists.

**Figure 5:**
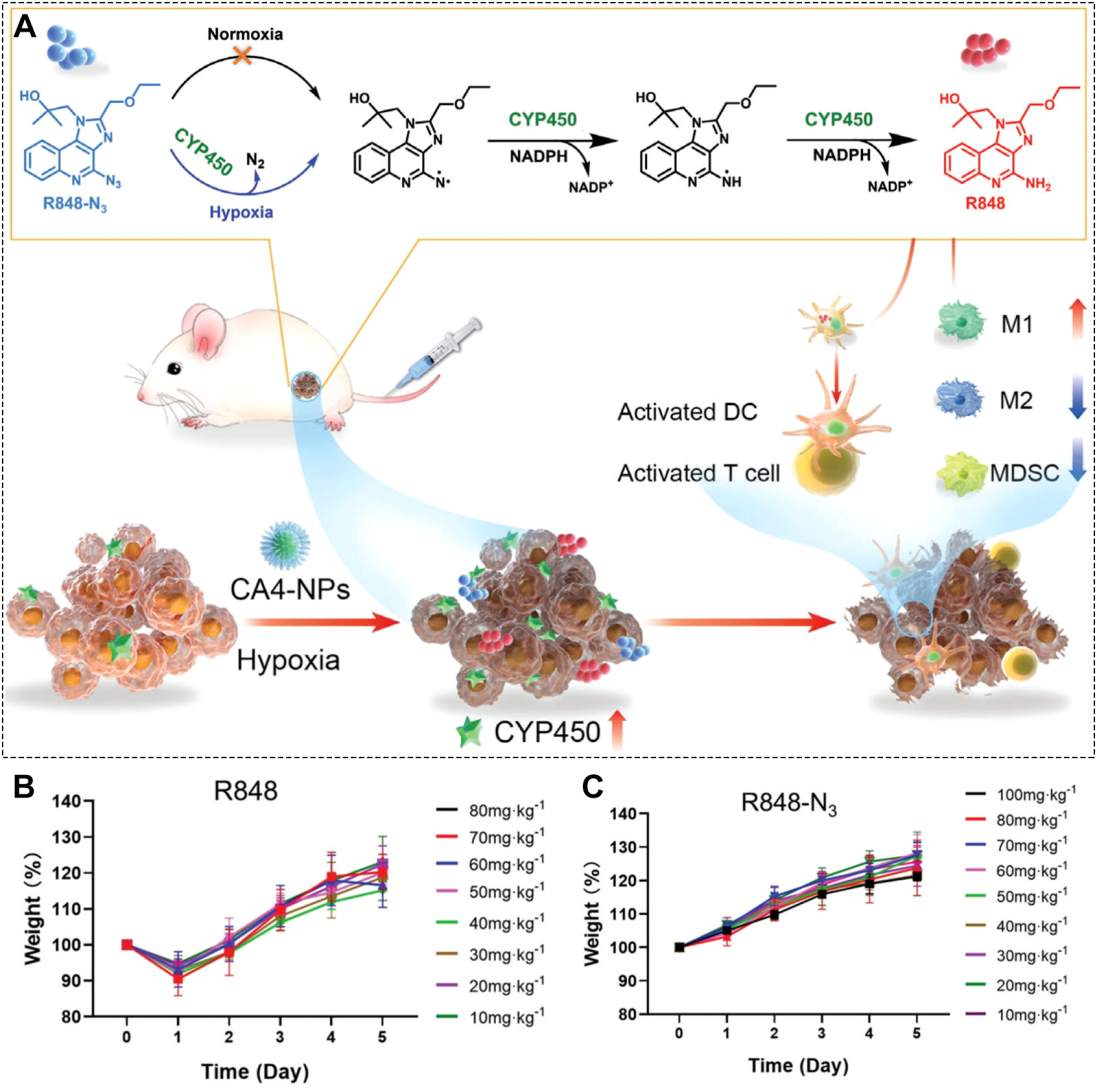
**(A)** Synthesis and activation mechanism of the R848-N_3_ prodrug. **(B)** Body weight changes in mice after R848 administration. **(C)** Body weight changes in mice after R848-N_3_ prodrug administration. Reproduced with permission from (84). Copyright (2023), Wiley-VCH GmbH.

The core idea of prodrug strategies is to temporarily “mask” the active molecule in a way that can be reversed only by specific signals present in the tumor. One effective approach for masking R848’s key aromatic amine group is to convert it into an azide group. The azide moiety is stable in normal tissues but can be selectively reduced back to the active amine by cytochrome P450 enzymes that are upregulated in the hypoxic microenvironment of many solid tumors. This reactivation restores R848’s immune-stimulating activity precisely where it is needed. Leveraging this mechanism, Liu and colleagues synthesized a small-molecule prodrug called R848-N_3_ (**Figure 5A**) (84). This compound remains inactive during circulation but is efficiently converted back to R848 specifically within tumor tissue. To further enhance tumor hypoxia, and thereby boost selective activation, the authors co-administered CA4-NPs. These nanoparticles disrupt immature tumor blood vessels, worsening hypoxia and creating more favorable conditions for R848-N_3_ reduction (**Figure 5A**). This azide-based strategy effectively reduced the risk of systemic inflammation caused by uncontrolled R848 activation. In toxicity studies, mice treated with free R848 showed significant weight loss, a common sign of systemic toxicity (**Figure 5B**). In contrast, mice receiving R848-N_3_ maintained stable body weight throughout the experiment (**Figure 5C**). These results clearly demonstrate that the azide masking strategy successfully suppresses R848’s off-target toxicity while preserving its therapeutic potential at the tumor site.

**Figure 6:**
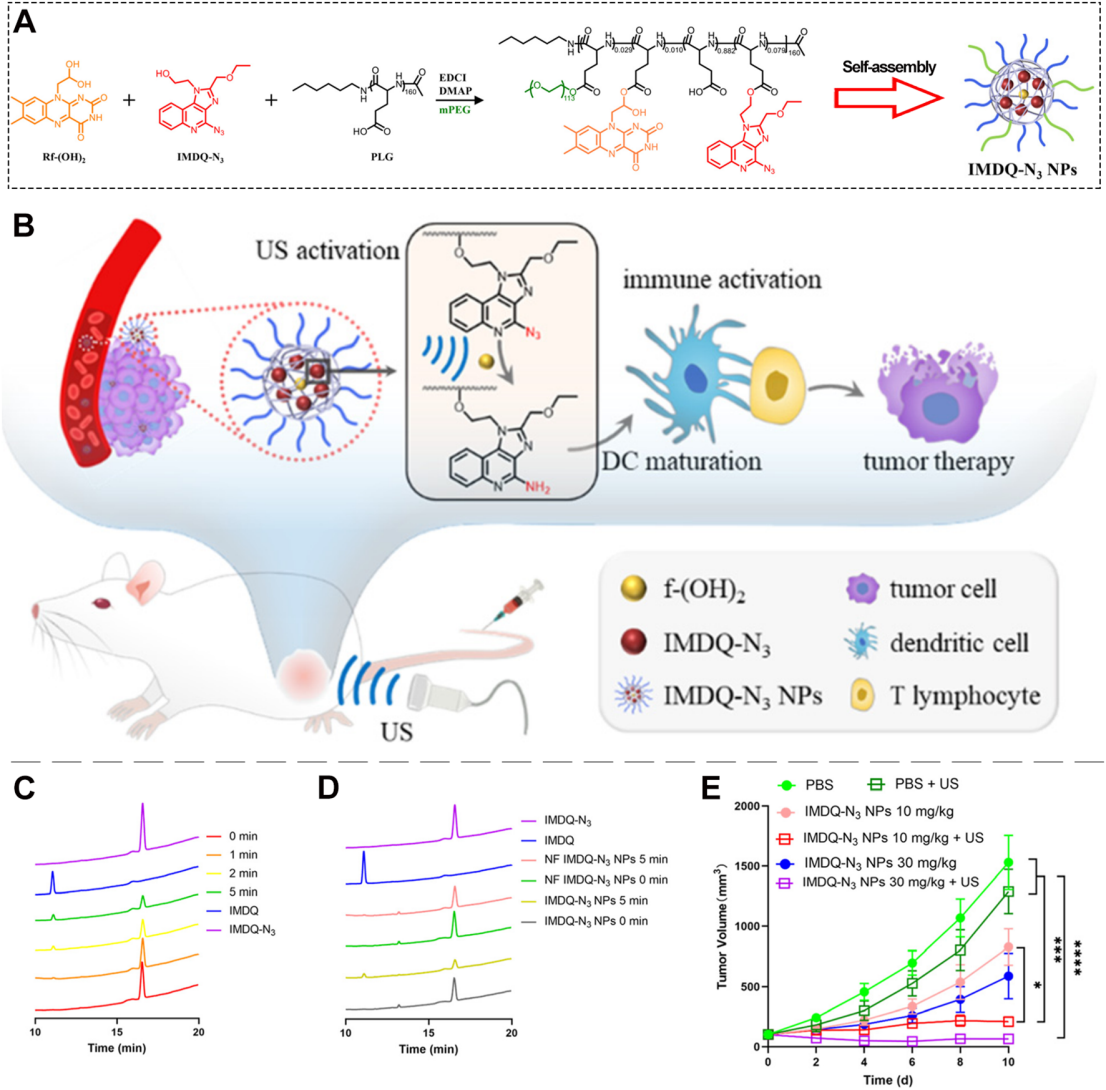
**(A)** Preparation of IMDQ-N_3_ nanoparticles (NPs). **(B)** Schematic illustration of IMDQ-N_3_ NPs-mediated immunotherapy. **(C)** Ultrasound-triggered generation of IMDQ in the absence of sonosensitizer. **(D)** Ultrasound-triggered generation of IMDQ in the presence of sonosensitizer. **(E)** Tumor volume changes in mice following IMDQ-N_3_ NP administration with ultrasound activation. *p <0.05, ***p <0.001, ****p <0.0001. Reproduced with permission from (89). Copyright (2025), The Royal Society of Chemistry.

Compared to endogenous triggers, such as the hypoxic or enzymatic conditions inside tumors, ultrasound offers superior spatiotemporal control for activating prodrugs (95–99). It also penetrates deep into tissues and works reliably regardless of tumor heterogeneity (100–104). When applied to tissue, ultrasound generates cavitation bubbles that split water molecules, producing hydrogen radicals (105–110). These hydrogen radicals can efficiently reduce azide groups back to active amines, making ultrasound an ideal tool to activate azide-masked prodrugs like IMDQ-N_3_. Taking advantage of this mechanism, Luo and colleagues developed an ultrasound-responsive system to activate IMDQ-N_3_. They co-conjugated both IMDQ-N_3_ and a sonosensitizer (riboflavin) onto a PEG-PLG polymer, which self-assembled into micellar nanoparticles (**Figure 6A**) (89). Their experiments showed that, under ultrasound irradiation and in the presence of riboflavin, IMDQ-N_3_ was efficiently converted into active R848, triggering a strong antitumor immune response (**Figure 6B**). This conversion was confirmed by HPLC analysis. In the control group without riboflavin, only 1.1% of IMDQ was generated (**Figure 6C**). But in the group containing the sonosensitizer, the conversion rate jumped to 13.7% (**Figure 6D**), demonstrating that riboflavin significantly enhances ultrasound-driven activation. In animal studies, this micelle system effectively suppressed tumor growth when activated by ultrasound (**Figure 6E**). Importantly, activation depended on both the external stimulus (ultrasound) and the built-in sonosensitizer, ensuring precise control. By using an external physical trigger to turn on the prodrug only at the tumor site, this approach offers a promising new strategy for the preclinical development of safer and more controllable TLR7/8 agonist therapies.

**Figure 7:**
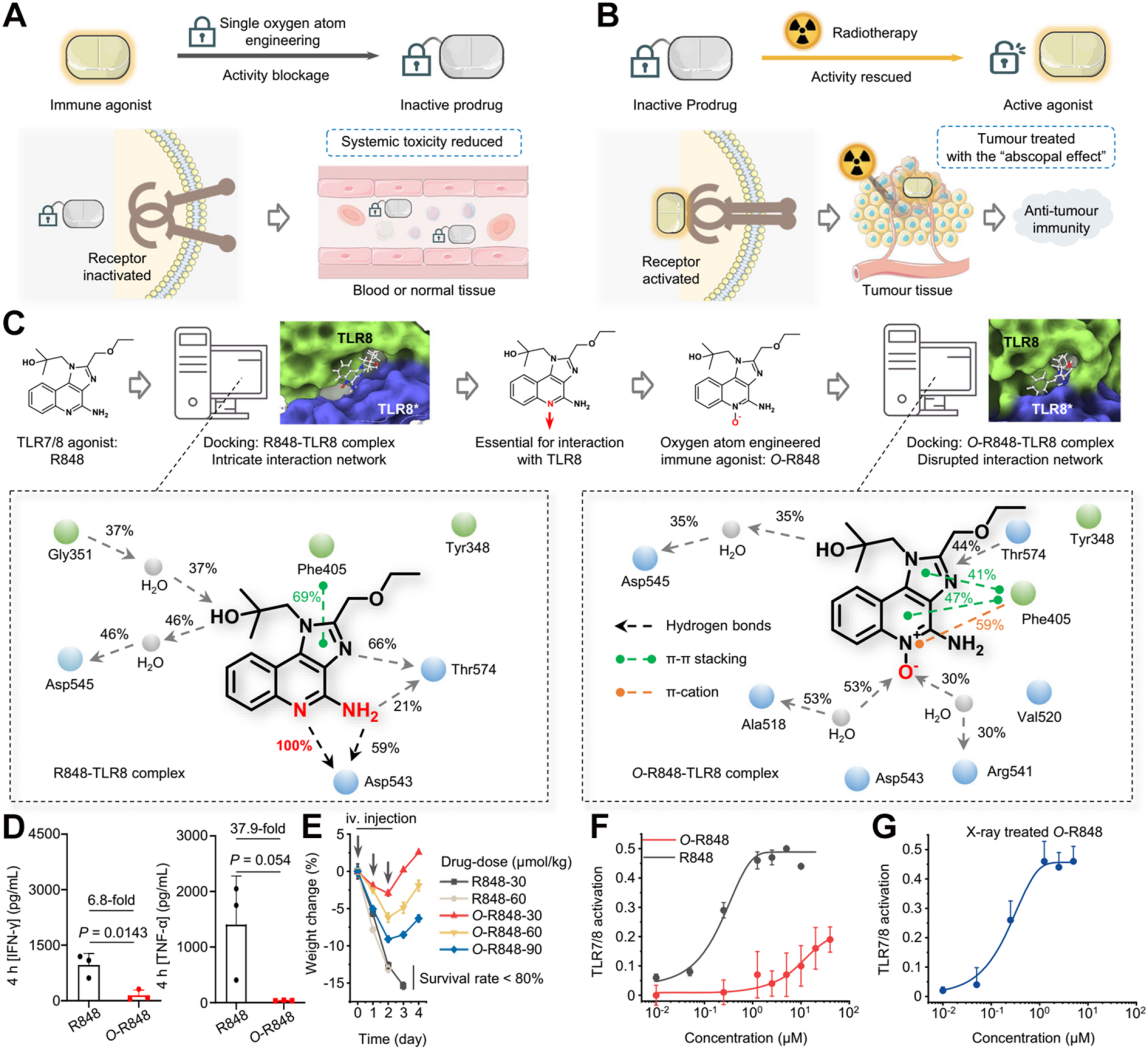
**(A)** Schematic illustration of R848 prodrug design via single oxygen atom masking. **(B)** Schematic of radiotherapy-triggered activation of O-R848 prodrug. **(C)** Computational design of the O-R848 prodrug. **(D)** Toxicity assessment of R848 versus O-R848 prodrug. **(E)** Body weight changes in mice after intravenous administration of R848 or O-R848. **(F)** Comparison of TLR7/8 activation potency between R848 and O-R848. **(G)** Mechanism of O-R848 activation under radiotherapy. Reproduced with permission from (86). Copyright (2025), Springer Nature.

In addition to masking the aromatic amine, Liu and colleagues discovered another clever way to silence R848’s activity: by adding a single oxygen atom at a critical site on the molecule. This small modification created a prodrug (named O-R848) whose potency dropped dramatically, the EC_50_ value was over 4,000 times higher than that of native R848 (**Figure 7A**) (86). This strategy effectively shut down R848’s immune-stimulating function, greatly reducing the risk of systemic toxicity after administration. Importantly, the “mask” can be removed on demand. When exposed to X-ray radiation, O-R848 undergoes a clean chemical transformation that removes the added oxygen atom, restoring the original R848 structure and its full immunostimulatory activity (**Figure 7B**). This design was first identified through computational docking studies and later validated experimentally (**Figure 7C**). To compare safety, the authors injected either R848 or O-R848 into mice via tail vein and measured levels of pro-inflammatory cytokines in the blood (**Figure 7D**) as well as changes in body weight (**Figure 7E**). The results clearly showed that O-R848 caused far less inflammation and no significant weight loss, confirming its lower systemic toxicity. Further assays confirmed that the EC_50_ of O-R848 was indeed ~4,000-fold higher than that of R848 (**Figure 7F**), proving the effectiveness of this chemical masking approach. When tumors were irradiated with X-rays, O-R848 was efficiently converted back to active R848 at the tumor site, triggering strong local immune activation (**Figure 7G**). Radiotherapy is a highly tissue-penetrating therapeutic modality widely used for treating various diseases and activating nanomedicine-based drug delivery systems (111–116). It can be harnessed to activate prodrugs in deep-seated tissues (117–124). This radiation-activated prodrug strategy in this work offers a new and practical path to harness the power of R848 while avoiding its dose-limiting toxicities, bringing it one step closer to clinical use.

## 4 Chemical design of prodrugs and diverse activation mechanisms

**Figure 8:**
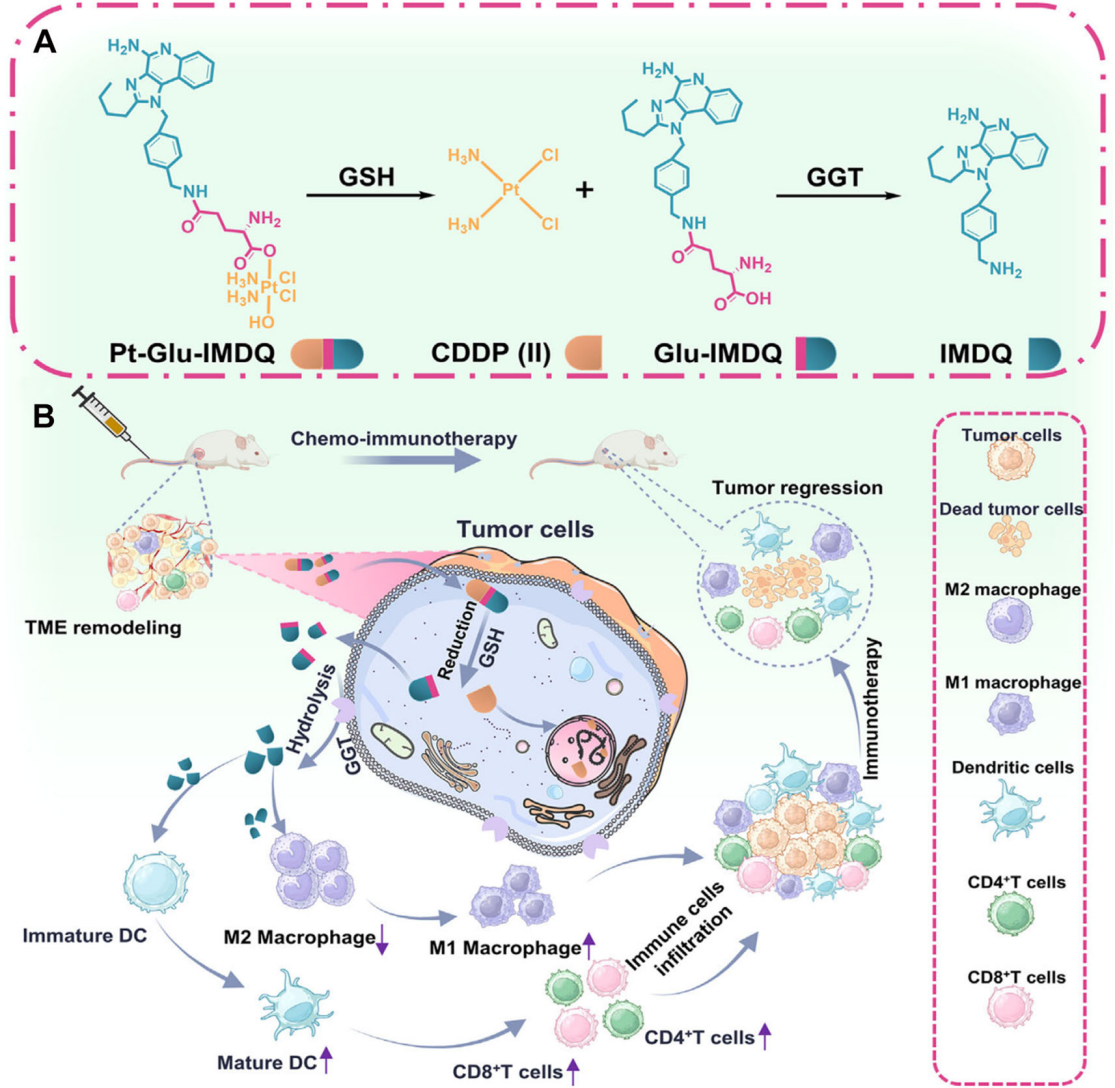
**(A)** Dual activation mechanism of Pt-Glu-IMDQ. **(B)** Schematic illustration of Pt-Glu-IMDQ prodrug–mediated combined chemo-immunotherapy for antitumor effects. Reproduced with permission from (62). Copyright (2025), Wiley-VCH GmbH.

The success of any prodrug strategy ultimately hinges on precise molecular design. Understanding how to design such prodrugs is therefore critically important. Numerous studies have shown that tumor-specific biochemical cues, such as overexpressed enzymes or acidic pH, can serve as “keys” to unlock carefully engineered prodrug “locks” (125–128). This approach enables selective activation only within the tumor microenvironment (129–132). For example, Mao and colleagues developed a dual-controlled prodrug by linking a platinum-based chemotherapeutic agent to a TLR7/8 agonist (IMDQ) via a γ-glutamyl linker (**Figure 8A**) (62). This molecule features two sequential “locks.” The researchers found that the prodrug is first cleaved by high levels of glutathione (GSH), a hallmark of many tumor cells. This step releases an intermediate that is then recognized and cut by γ-glutamyl transpeptidase (GGT), an enzyme often overexpressed on tumor cell membranes. Only after both steps are completed are active cisplatin and IMDQ simultaneously released. This tandem activation achieves two goals at once: it couples chemotherapy with immune stimulation, and it keeps the potent IMDQ masked until it reaches the tumor, effectively isolating its toxicity. As a result, the system delivers strong synergistic antitumor effects (**Figure 8B**). This work exemplifies an elegant, multi-signal-responsive design that leverages the unique biology of tumors to ensure precise and coordinated drug release.

In addition to targeting extracellular enzymes, another common and effective activation strategy takes advantage of the acidic environment inside cellular compartments, particularly endosomes and lysosomes. For example, Aichhorn and colleagues developed a macromolecular prodrug by attaching an IMDQ derivative to a biodegradable polymer, poly(organo)phosphazene, using a pH-sensitive hydrazone bond (87). This design relies on the low pH found in endosomes and lysosomes to break the hydrazone linker and release the active drug. Because this acidic environment only exists after the prodrug has been taken up by cells, activation occurs primarily inside antigen-presenting cells that have internalized the construct. This approach significantly improves both targeting precision and safety, ensuring that immune stimulation happens where it’s needed most, while minimizing off-target effects.

## 5 Delivery system design: from nanocarriers to long-acting depots

Smart molecular design gives prodrugs the potential to respond intelligently to specific triggers. However, this potential can only be fully realized if the prodrugs are efficiently delivered to the right tissues or cells, and work together with other immune-modulating agents. This is where delivery systems come in. To bridge the gap between clever chemistry and real-world therapeutic impact, the researchers should move beyond molecules alone and turn to materials science and pharmaceutics. Designing effective carriers to “arm” and “deliver” these prodrugs is therefore crucial. Such systems not only protect the prodrug during circulation but also enhance its accumulation at the target site, control its release, and enable combination strategies, making them indispensable for next-generation immunotherapy.

**Figure 9:**
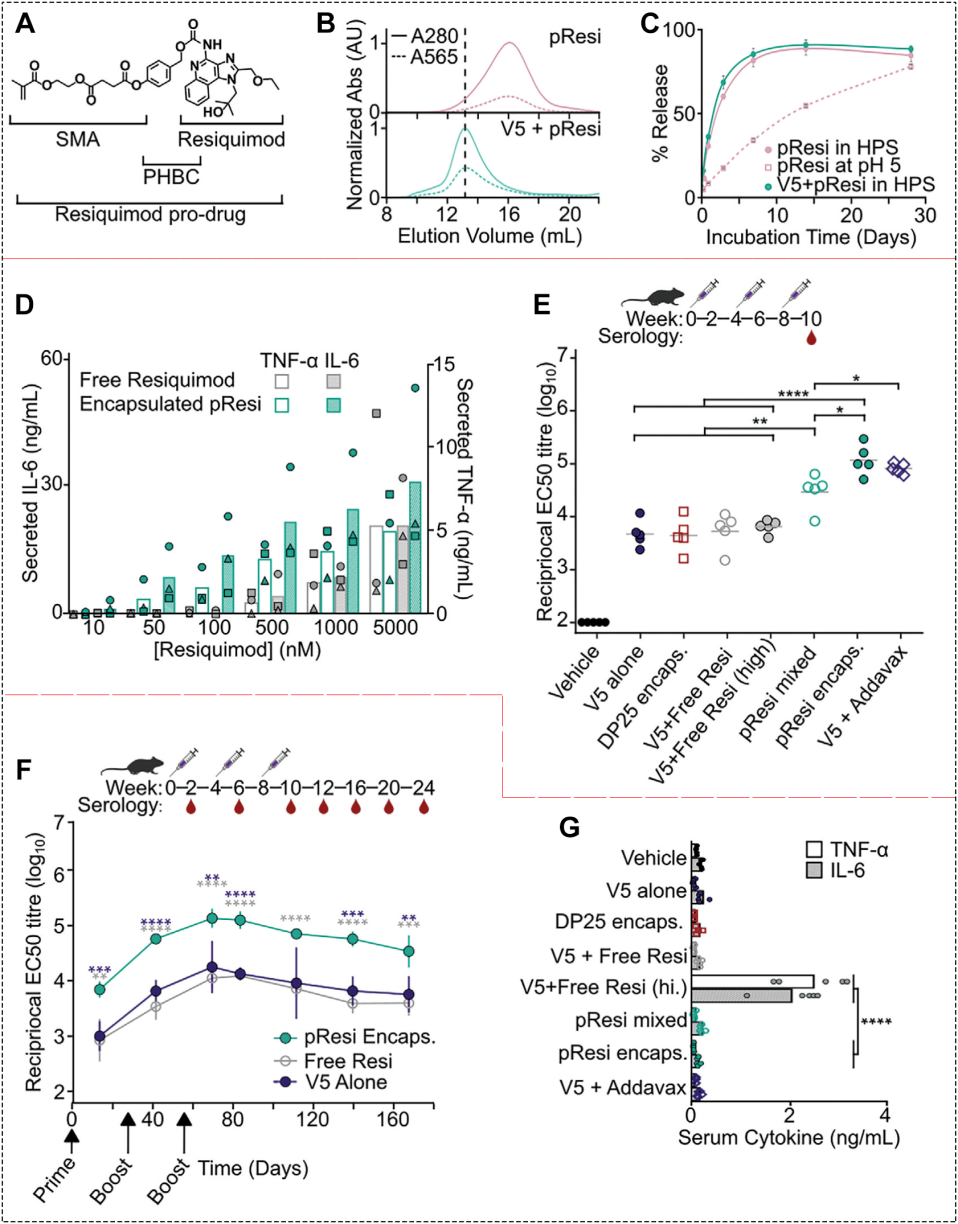
**(A)** Chemical structure of the R848 prodrug. **(B)** Encapsulation efficiency of R848 prodrug nanoparticles assessed by rhodamine absorbance. **(C)** Release profile of the encapsulated R848 prodrug. **(D)** Comparison of IL-6 and TNF-α induction by free versus encapsulated R848 prodrug. **(E)** Immune evaluation in mice across different treatment groups. *p <0.05, **p <0.01, ****p <0.0001. **(F)** Duration of immune responses induced by free R848 prodrug, encapsulated R848 prodrug, and protein-based delivery. **(G)** Systemic toxicity assessment via serum cytokine levels in different treatment groups. Reproduced with permission from (92). Copyright (2024), Wiley-VCH GmbH.

Even the most refined prodrug molecules need advanced delivery systems to achieve efficient transport, targeted accumulation, controlled release, and functional synergy (133–138). To deliver TLR7/8 agonist prodrugs effectively, researchers have developed a variety of nano- and micrometer-scale platforms, such as nanoparticles, nanogels, liposomes, and polymeric vesicles. For instance, Herpoldt and colleagues engineered a protein-based nanoparticle capable of co-loading multiple therapeutic agents (92). They successfully encapsulated an R848 prodrug (named PHBC) into this carrier (**Figure 9A**). Evidence from size-exclusion chromatography showed a clear shift in elution volume, confirming effective encapsulation (**Figure 9B**). Each protein nanoparticle carried approximately 100 PHBC molecules. Importantly, the protein shell did not trigger premature drug release during circulation (**Figure 9C**), ensuring stability in the bloodstream. In cellular studies, the nanoparticle-formulated prodrug was taken up more efficiently by immune cells. This led to significantly higher cytokine production compared to free prodrug (**Figure 9D**). In mice, the protein-encapsulated R848 prodrug induced a stronger immune response than all control groups (**Figure 9E**). Moreover, the immune activation lasted longer (**Figure 9F**), suggesting sustained activity at the target site. To assess the impact of carrier encapsulation on systemic toxicity, serum cytokine levels were measured 1 h after immunization. As shown in **Figures 9G**, free resiquimod (20 μg) induced elevated levels of TNF-α and IL-6, reflecting its inherent systemic cytokine-mediated toxicity as an immune adjuvant. In contrast, the encapsulated formulation resulted in significantly lower cytokine levels. Toxicity tests further confirmed that encapsulation dramatically reduced systemic side effects (**Figure 9G**). Together, these results show that using protein nanoparticles to deliver R848 prodrugs offers dual benefits: it markedly lowers toxicity while significantly boosting immunogenicity, turning a potent but risky molecule into a safer and more effective therapeutic agent.

**Figure 10:**
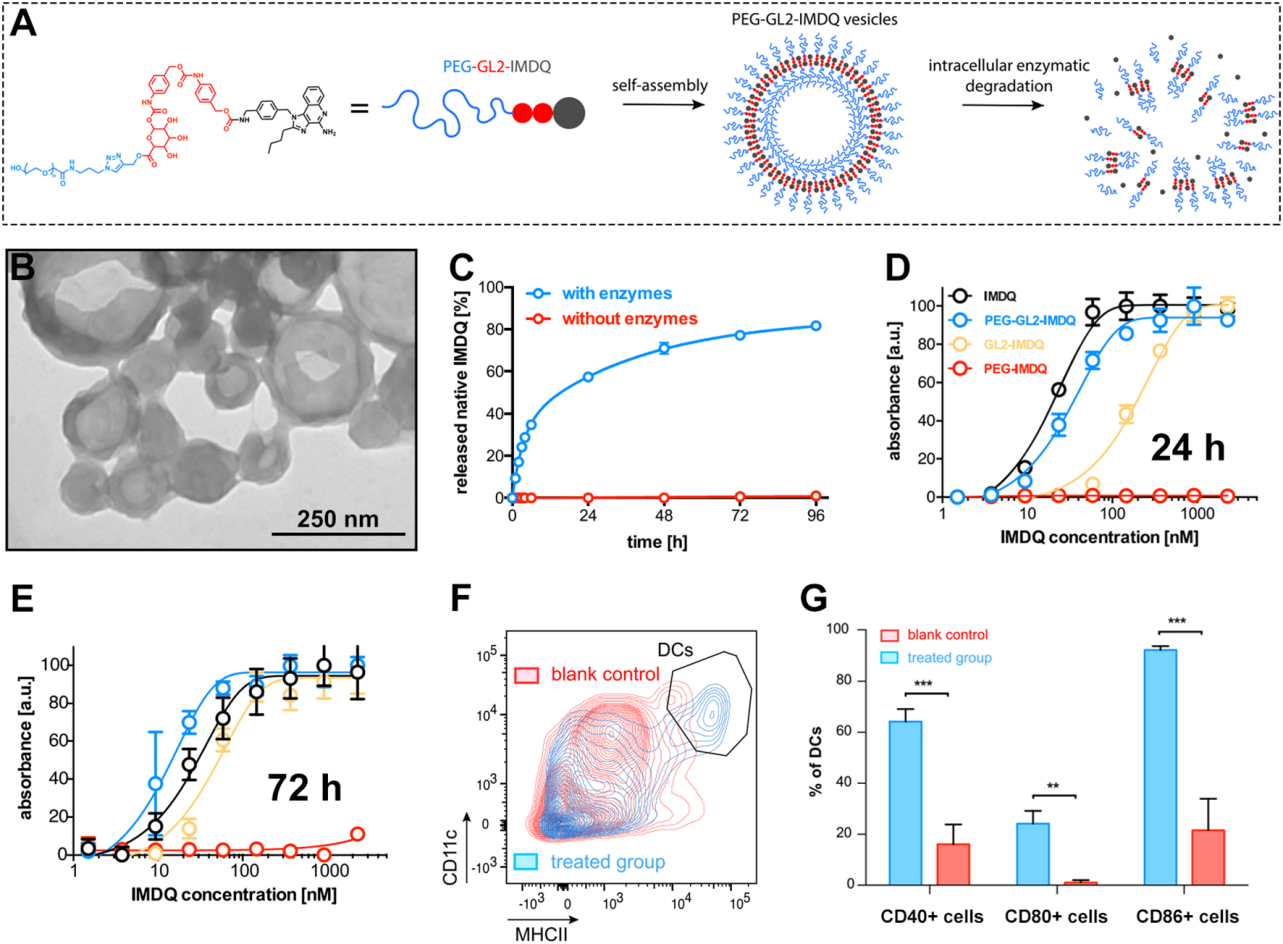
**(A)** Schematic of PEG-GL2-IMDQ vesicle preparation and enzymatic degradation. **(B)** TEM image of PEG-GL2-IMDQ vesicles. **(C)** IMDQ release profile from PEG-GL2-IMDQ vesicles. **(D)** *In vitro* TLR7/8 activation by different formulations at 24 h **(E)** *In vitro* TLR7/8 activation by different formulations at 72 h **(F)** Dendritic cell (DC) activation by PEG-GL2-IMDQ vesicles. **(G)** Comparison of DC maturation marker expression across treatment groups. **p <0.01, ***p <0.001. Reproduced with permission from (94). Copyright (2020), American Chemical Society.

Amphiphilic polymer–prodrug conjugates that self-assemble into vesicles have also shown excellent drug-loading capacity and controlled release properties. For example, Shi and colleagues linked an amphiphilic polymer to a TLR7/8 agonist (IMDQ), creating a conjugate called PEG-GL2-IMDQ (**Figure 10A**) (94). This molecule spontaneously formed vesicles in aqueous solution (**Figure 10A**), with an average size of about 200 nm (**Figure 10B**). Once internalized by cells, the conjugate was efficiently cleaved by endosomal enzymes, releasing active IMDQ molecules (**Figure 10C**). *In vitro* assays using immune cells confirmed that the PEG5k-GL2-IMDQ vesicles retained strong TLR-activating activity (**Figures 10D, E**). Remarkably, after 72 h of incubation, the vesicle formulation even outperformed free (native) IMDQ in stimulating immune responses (**Figure 10E**), highlighting the sustained and enhanced activity enabled by this delivery system. In mouse studies, the vesicles effectively activated dendritic cells (DCs) (**Figure 10F**). This was evidenced by significantly increased expression of key maturation markers, CD40, CD80, and CD86, on the DC surface (**Figure 10G**). Notably, this work provided the first clear evidence that IMDQ delivered via a vesicular carrier can exhibit biological activity equal to or even greater than that of the free small molecule. This finding is highly significant, as it offers a promising new approach to boost the potency and therapeutic utility of IMDQ-based immunotherapies.

In addition, Lu and colleagues developed a nanosuspension by formulating an R848 prodrug with hyaluronic acid (71). This system enabled efficient delivery of the prodrug to the target site. The formulation effectively prevented the systemic toxicity typically caused by free R848. At the same time, it significantly prolonged drug release, extending the duration of action without compromising TLR-agonist activity. This approach thus balances safety, sustained release, and immunostimulatory potency in a single delivery platform.

**Figure 11:**
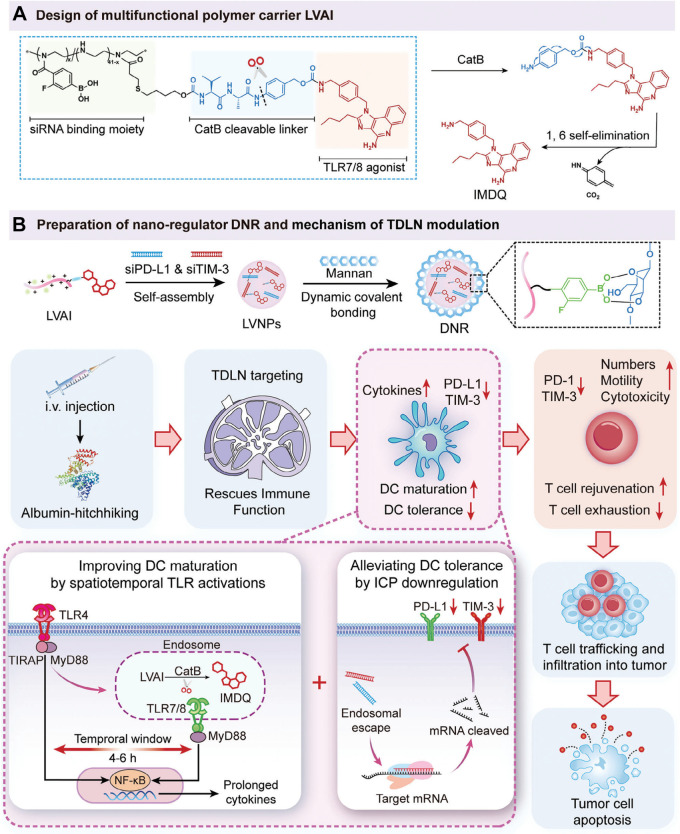
**(A)** Synthesis and release mechanism of the IMDQ prodrug. **(B)** Preparation of the nanoregulator and its multimodal combinatorial antitumor immunotherapy mechanism. Reproduced with permission from (90). Copyright (2024), Wiley-VCH GmbH.

One key advantage of advanced delivery systems is their ability to integrate multiple functional components, enabling synergistic immune modulation. For example, Tain and colleagues developed a spatiotemporally controlled nanoregulator that co-delivers a TLR7/8 agonist, a TLR4 agonist, and siRNA targeting immune checkpoint proteins (ICPs) (90). This system simultaneously reshapes both the TDLNs and the tumor microenvironment, leading to potent combinatorial immunotherapy. The team first synthesized a cathepsin B–cleavable IMDQ prodrug (named LVAI) via conjugation chemistry (**Figure 11A**). Thanks to its positive charge, this prodrug could electrostatically bind negatively charged siRNA (**Figure 11B**). Next, they coated the surface of the self-assembled nanoparticles with mannan, a natural TLR4 agonist (**Figure 11B**). This sugar polymer enabled the final nanocomplex (named DNR) to hitch a ride on circulating albumin after intravenous injection, directing it selectively to TDLNs (**Figure 11B**), a process known as the “albumin-hitchhiking” mechanism. Once in the lymph nodes, DNR released both TLR agonists. The TLR4 and TLR7/8 signals worked together to strongly and persistently activate DCs (**Figure 11B**). Meanwhile, the co-delivered siRNA silenced ICPs in DCs, reducing their tolerance and further boosting their stimulatory capacity (**Figure 11B**). As a result, T-cell priming and activation were dramatically enhanced, leading to robust antitumor immunity. This strategy demonstrates how rational design and precise integration of multiple agents into a single nano-platform can unlock powerful synergy, turning separate therapeutic elements into a coordinated immune-activating program.

**Figure 12:**
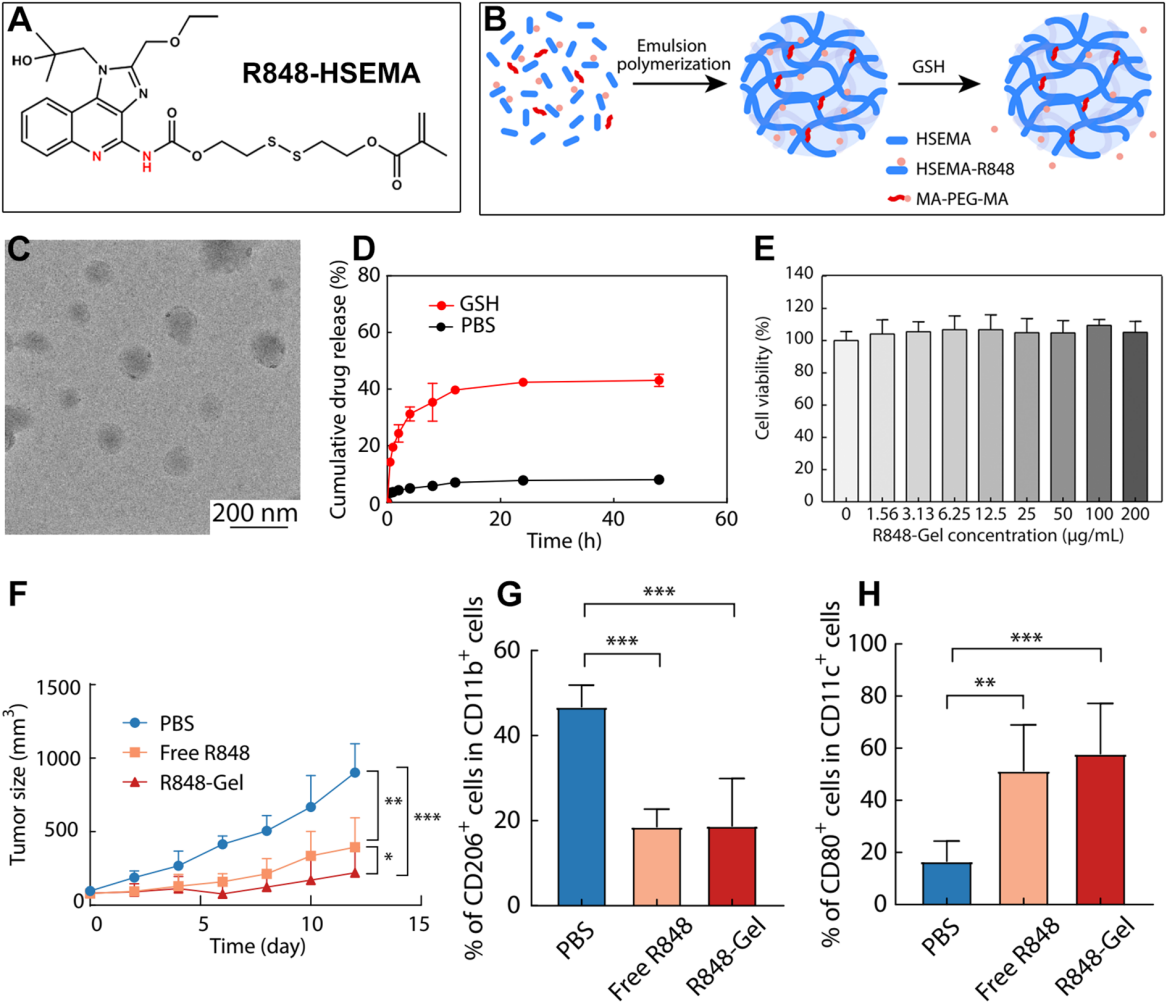
**(A)** Chemical structure of the R848 prodrug. **(B)** Schematic illustration of nanogel formation. **(C)** TEM image of the nanogels. **(D)** Release of R848 from nanogels in response to GSH. **(E)** Biocompatibility assessment of the nanogels. **(F)** *In vivo* antitumor efficacy of the nanogels. *p <0.05, **p <0.01, ***p <0.001. **(G, H)** Changes in tumor-infiltrating immune cells after treatment: **(G)** CD206^+^ cells and **(H)** CD80^+^ cells. *p <0.05, **p <0.01, ***p <0.001. Reproduced with permission from (88). Copyright (2022), Chinese Chemical Society and Institute of Chemistry, CAS.

Hydrogel are highly versatile drug carriers thanks to their three-dimensional crosslinked network structure (139–141). They can load multiple therapeutic agents, either by physical entrapment or chemical conjugation, making them adaptable to a wide range of diseases (142–144). Nanogels are nanoscale hydrogels that have been extensively studied in recent years. Their nanoscale size enhances passive tumor targeting through the EPR effect, which helps reduce off-target toxicity in healthy tissues. Moreover, the gel network can swell or shrink in response to tumor-specific cues, such as pH, temperature, or enzymes, enabling precise, stimulus-triggered drug release and improving treatment efficacy. Because of these advantages, nanogels have become one of the most promising platforms in controlled drug delivery. Building on this concept, Du and colleagues developed a redox-responsive nanogel encapsulating an R848 prodrug (88). First, they linked R848 to a small molecule containing a disulfide bond (HSEMA), creating R848-HSEMA (**Figure 12A**). This conjugate was then used in an emulsion polymerization to form nanogels (**Figure 12B**). Transmission electron microscopy (TEM) confirmed that the particles were approximately 100 nm in size (**Figure 12C**). In the presence of GSH, which is abundant inside tumor cells, the disulfide bonds broke, disrupting the gel network and efficiently releasing active R848 (**Figure 12D**). The nanogel also showed excellent biocompatibility *in vitro* (**Figure 12E**). More importantly, in a mouse model of breast cancer, it successfully released R848 at the tumor site and significantly suppressed tumor growth (**Figure 12F**). The authors further analyzed immune cell infiltration in treated tumors. Results showed a clear decrease in immunosuppressive M2-type macrophages and strong activation of APCs (**Figures 12G, H**). This shift helped reprogram the tumor microenvironment from immunosuppressive to immunostimulatory. Overall, this redox-responsive nanogel offers a new and effective approach for the sustained, tumor-selective release of R848, balancing safety, control, and potent immune activation.

## 6 Integrated therapeutic strategies and preclinical/clinical translation

**Figure 13:**
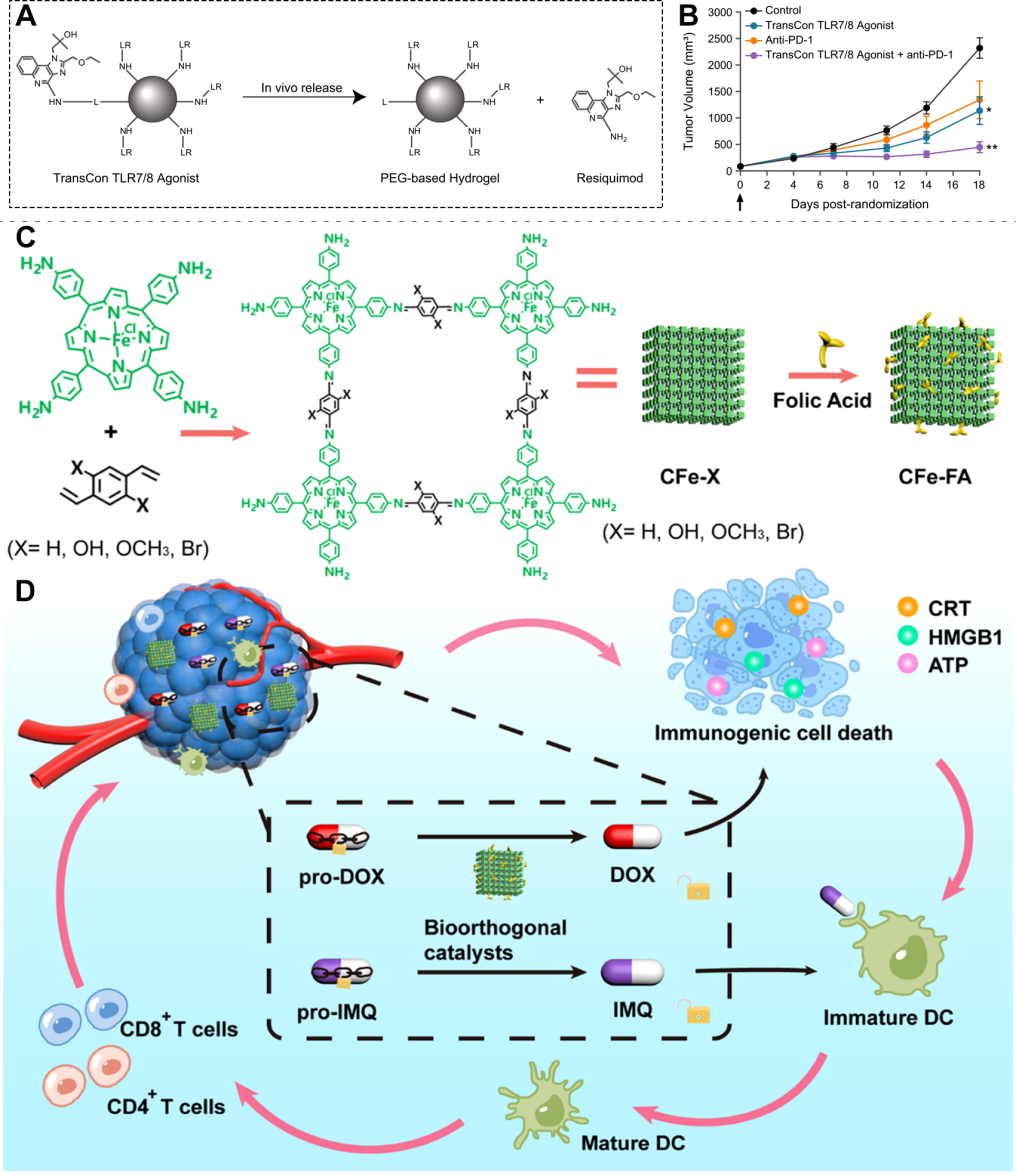
**(A)** Schematic of TransCon TLR7/8 agonist release. **(B)** *In vivo* antitumor efficacy of TransCon TLR7/8 agonist combined with anti-PD-1 in mice. *p <0.05, **p <0.01. Reproduced with permission from (91). Copyright (2022), BioMed Central. **(C)** Structure and synthesis of the CFe-FA carrier. **(D)** Schematic illustration of CFe-FA–mediated combined chemo-immunotherapy. Reproduced with permission from (93). Copyright (2023), American Chemical Society.

Once safe, smart, and multifunctional prodrug delivery systems are successfully built, their true value must be tested in real therapeutic settings. Indeed, combining prodrug-based delivery with other mainstream cancer therapies, such as immune checkpoint inhibitors, chemotherapy, or cytokine therapy, has proven highly effective and promising. These advanced strategies are already delivering breakthrough results in complex combination regimens and early-stage clinical translation, paving the way for broader use of TLR7/8 agonists in oncology. A notable example is the TransCon TLR7/8 agonist developed by Punnonen and colleagues, now being evaluated in clinical trials (**Figure 13A**) (91). This system uses a proprietary hydrogel-based linker technology that enables a single intratumoral injection to release the active drug slowly over several weeks. This sustained release dramatically extends local drug exposure while minimizing systemic toxicity. The authors evaluated the immunotherapeutic efficacy of TransCon TLR7/8 agonist in CT26 tumor-bearing mice via intratumoral injection. In both preclinical models and early clinical studies, the agent showed strong antitumor activity, whether used alone or in combination with anti-PD1 (**Figure 13B**). This approach marks a major advance in prodrug engineering and clinical translation, demonstrating how smart formulation design can turn a potent but challenging immunostimulant into a practical and effective cancer therapy.

Beyond improving how existing drugs are delivered, the most forward-looking strategies aim to create entirely new therapeutic paradigms, by deeply integrating prodrug activation with other treatments at the molecular level. Doxorubicin exerts potent anticancer effects but can cause cardiotoxicity and drug resistance (145–148). Therefore, developing doxorubicin-loaded delivery systems conjugated with targeting ligands such as folic acid is highly desirable (149–152). Moreover, combination therapy can help mitigate the adverse effects associated with doxorubicin monotherapy (153, 154). Based on this, Sun and colleagues developed a novel “bioorthogonal catalysis–activated *in situ* vaccine” using the COF as the carrier (**Figures 13C, D**) (93). In this system, functional nanocatalysts (Fe^2+^) are delivered to the tumor. Once there, they trigger two key reactions simultaneously: they convert a prodrug form of doxorubicin into its active chemotherapy form, and they also activate a prodrug version of imiquimod, a TLR7 agonist. This dual activation ensures that immunogenic cell death (from chemotherapy) and adjuvant immune stimulation (from imiquimod) occur in the same place and at the same time. The precise spatiotemporal coupling maximizes antitumor immunity while avoiding systemic side effects (**Figure 13D**). *In vivo* studies in 4T1 tumor-bearing models further demonstrated that intraperitoneal co-administration of DOX and IMQ prodrugs conferred significantly enhanced antitumor efficacy. This approach represents the cutting edge of prodrug technology, where smart chemistry, nanocatalysis, and immunotherapy converge to create a powerful and self-amplifying cancer vaccine directly inside the tumor.

## 7 Conclusion

Over the past few years, tumor immunotherapy based on R848 prodrugs has undergone a significant paradigm shift. The core achievement is not merely the creation of new drug molecules or delivery vehicles. Rather, it lies in the successful development of a hierarchical, spatiotemporally precise control framework, one that fundamentally challenges the long-held belief that potent immune agonists cannot be safely administered systemically. This new paradigm operates on three interconnected levels. At the molecular level, researchers have engineered “smart” prodrugs that remain inactive until triggered by specific cues in the tumor microenvironment, such as hypoxia, overexpressed enzymes, acidic pH, or high reducing potential, or by external physical stimuli like ultrasound or radiotherapy. This transforms R848 from a constantly “on” molecule into a dormant agent that awakens only upon receiving a pre-defined signal. At the delivery engineering level, diverse platforms, from nanoparticles to macroscopic depots, have been developed. These systems do more than just deliver prodrugs to tumors and sustain their release. They function as integrated therapeutic hubs capable of co-delivering antigens, siRNA, chemotherapeutics, or even nanocatalysts, enabling coordinated multimodal therapy. At the treatment strategy level, this approach has proven highly synergistic with immune checkpoint inhibitors and conventional chemo/radiotherapy. By coupling localized immune activation with systemic immune engagement, it can generate robust, long-lasting antitumor immune memory. Thus, current research has moved beyond simply reducing toxicity. It now enters a new era: actively programming immune responses through multi-layered engineering.

Despite this progress, translating this promising paradigm into broad clinical use faces three deep challenges. First, the biggest hurdle is the extreme heterogeneity of human tumors and the complexity of biological barriers. Activation strategies relying on hypoxia, specific enzymes, or pH are limited by how unevenly these signals appear, not just between patients, but even within a single tumor. Moreover, the EPR effect, often assumed to guide nanoparticle delivery, is highly variable and unreliable in human cancers. Most current “controlled release” systems still offer only coarse regulation. Precisely matching drug release kinetics to the optimal timing of immune activation, avoiding either excessive local inflammation or T-cell exhaustion, remains an unsolved fine-control problem. Second, translational challenges are even steeper. The gap between mouse models and human patients is vast: murine immune systems and tumor models poorly replicate human complexity. Many sophisticated nanoplatforms struggle with scalable manufacturing, batch-to-batch consistency, and long-term safety. Local delivery methods like intratumoral injection are impractical for deep-seated or metastatic tumors, while systemic delivery still lacks sufficient targeting precision. Third, from a regulatory and mechanistic perspective, we lack standardized frameworks to evaluate complex combination products, those integrating prodrugs, cleavable linkers, and functional carriers, in terms of pharmacokinetics, toxicology, and immunogenicity. More importantly, our understanding of how these systems reshape the dynamic immune landscape remains superficial. Most studies focus on final tumor shrinkage, but we still know little about how prodrug strategies precisely influence the spatial and temporal behavior of specific immune subsets, such as dendritic cell subtypes or T-cell differentiation states, or how they shape the quality of long-term immune memory.

Looking ahead, overcoming these barriers will require deep interdisciplinary integration and disruptive innovation. To tackle scientific challenges, next-generation systems should be adaptive. Examples include logic-gated prodrugs that respond only when multiple tumor signals are present simultaneously, or externally regulated platforms (e.g., repeatable ultrasound or magnetic hyperthermia) that allow on-demand control despite tumor heterogeneity. AI-assisted design could optimize carrier shape, surface chemistry, and tumor-penetrating ability. Integrating diagnostic functions, such as real-time monitoring of drug release and immune activation, could enable truly personalized dosing. To address translational gaps, we must adopt more clinically predictive models: humanized mice, patient-derived organoids, or organ-on-chip systems. Delivery platforms should prioritize modularity, biodegradability, and chemical definition, moving toward “plug-and-play” systems like computationally designed protein nanoparticles that simplify manufacturing. Expanding treatment scope will also require non-invasive or minimally invasive strategies, such as cell-based carriers, biomimetic membrane coatings, or novel formulations for regional or cavity-directed delivery. To deepen mechanistic understanding, we need high-resolution tools, single-cell multi-omics, spatial transcriptomics, multiplexed imaging, to map the dynamic evolution of the tumor immune microenvironment under prodrug therapy. Such insights will reveal exactly how these systems convert “cold” tumors “hot” and reverse T-cell exhaustion, guiding rational combinations with various immunotherapy strategies. Moreover, in the studies discussed in this review, it is important to note that although local delivery aims to confine immune activation within the tumor microenvironment, potential “spillover effects” must be carefully considered, namely, that potent local stimulation may inadvertently trigger harmful systemic inflammation through cytokine release or immune cell trafficking. Nevertheless, a key advantage of prodrug-based strategies lies in their dual controllability: first, by restricting activation to the tumor site through microenvironmental or external triggers, systemic drug exposure is minimized; second, spatiotemporally precise release promotes antigen-specific T-cell expansion and memory formation, thereby eliciting beneficial systemic antitumor immunity rather than nonspecific inflammatory cascades. Future designs should further refine activation thresholds and release kinetics to maximize therapeutic systemic immune responses while avoiding adverse events such as cytokine release syndrome.

In summary, R848-based prodrug strategies have successfully opened a path toward safe and effective tumor immune activation. The future no longer lies in optimizing single molecules or carriers in isolation. Instead, it demands the seamless fusion of chemistry, materials science, engineering, immunology, and clinical medicine to build truly intelligent, adaptive, and clinically viable immunotherapy systems. The road is challenging, but the goal is clear: to deliver powerful immune stimulation like a precision-guided weapon, activating immunity exactly where and when it’s needed, while sparing the rest of the body. This vision will continue to drive breakthrough innovations and bring new hope to cancer patients.
